# Differentially Expressed MicroRNAs in Chondrocytes from Distinct Regions of Developing Human Cartilage

**DOI:** 10.1371/journal.pone.0075012

**Published:** 2013-09-09

**Authors:** Audrey McAlinden, Nobish Varghese, Louisa Wirthlin, Li-Wei Chang

**Affiliations:** 1 Department of Orthopaedic Surgery, Washington University, St Louis, Missouri, United States of America; 2 Department of Cell Biology and Physiology, Washington University, St Louis, Missouri, United States of America; 3 Department of Pathology and Immunology, Washington University, St Louis, Missouri, United States of America; University of California, San Diego, United States of America

## Abstract

There is compelling *in vivo* evidence from reports on human genetic mutations and transgenic mice that some microRNAs (miRNAs) play an important functional role in regulating skeletal development and growth. A number of published *in vitro* studies also point toward a role for miRNAs in controlling chondrocyte gene expression and differentiation. However, information on miRNAs that may regulate a specific phase of chondrocyte differentiation (i.e. production of progenitor, differentiated or hypertrophic chondrocytes) is lacking. To attempt to bridge this knowledge gap, we have investigated miRNA expression patterns in human embryonic cartilage tissue. Specifically, a developmental time point was selected, prior to endochondral ossification in the embryonic limb, to permit analysis of three distinct populations of chondrocytes. The location of chondroprogenitor cells, differentiated chondrocytes and hypertrophic chondrocytes in gestational day 54–56 human embryonic limb tissue sections was confirmed both histologically and by specific collagen expression patterns. Laser capture microdissection was utilized to separate the three chondrocyte populations and a miRNA profiling study was carried out using TaqMan® OpenArray® Human MicroRNA Panels (Applied Biosystems®). Here we report on abundantly expressed miRNAs in human embryonic cartilage tissue and, more importantly, we have identified miRNAs that are significantly differentially expressed between precursor, differentiated and hypertrophic chondrocytes by 2-fold or more. Some of the miRNAs identified in this study have been described in other aspects of cartilage or bone biology, while others have not yet been reported in chondrocytes. Finally, a bioinformatics approach was applied to begin to decipher developmental cellular pathways that may be regulated by groups of differentially expressed miRNAs during distinct stages of chondrogenesis. Data obtained from this work will serve as an important resource of information for the field of cartilage biology and will enhance our understanding of miRNA-driven mechanisms regulating cartilage and endochondral bone development, regeneration and repair.

## Introduction

Development of cartilage tissue begins by the condensation of mesenchymal stem cells (MSCs) and subsequent differentiation of these cells toward the chondrocyte lineage. Cells within the cartilage anlagen proliferate and generate an extracellular matrix (ECM) rich in type II collagen and proteoglycans, thus permitting cartilage growth and subsequent limb formation [1]. Cartilage of the growth plate serves as a template for long bone formation whereby proliferating chondrocytes terminally differentiate to become large hypertrophic cells that specifically synthesize type X collagen. Preceding apoptotic cell death, hypertrophic chondrocytes regulate matrix mineralization and blood vessel invasion resulting in cancellous bone formation by a process known as endochondral ossification [2]. Articular cartilage is found at the bone epiphyses lining our joints and functions to lubricate and distribute load within the joint. This hyaline cartilage is distinct from growth plate cartilage in that chondrocytes do not terminally differentiate toward hypertrophy and the mature tissue remains avascular [3]–[5]. Many genes encoding a wide range of molecules such as histone modifying proteins, transcription factors, growth factors, ECM components, ECM modifying enzymes, cell receptors etc are known to regulate specific stages of chondrocyte differentiation. Mutations in or ablation of such cartilage-regulatory genes are known to cause skeletal abnormalities, resulting in diseases such as chondrodysplasias (growth plate cartilage defects) or osteoarthritis (articular cartilage defects).

It has been estimated that as many as 60% of coding genes are regulated by the family of small, non-coding RNAs called microRNAs (miRNAs) [6]. This introduces another level of complexity into the mechanisms that control cellular functions for proper development of tissues and organs of the body, including those of the skeletal system. With respect to miRNA biogenesis, large primary (pri) miRNAs are first transcribed from intergenic regions of the genome, or from introns of coding genes, and then processed in the nucleus by a Drosha-containing protein complex to smaller, 70–100 nucleotide (nt) precursor (pre) miRNAs. Pre-miRNAs are transported from the nucleus to the cytoplasm by Exportin 5 and then further processed by another protein complex containing the enzyme Dicer to generate small (∼19–24 nt) imperfect double-stranded miRNA duplexes. These miRNA duplexes enter the RNA induced silencing complex (RISC) containing important proteins called Argonautes that guide interaction of either the 5p or 3p miRNA strand with its target mRNA sequence [7]. These target sequences are commonly located in 3′UTRs but there are increased reports that miRNAs can also bind to sequences within exons [8]. The overall effect of these miRNA-target gene interactions is to down-regulate protein expression via translational repression or mRNA degradation [9], [10].

Since their discovery in *C. elegans* in 1993, we now understand a lot about the physiological roles of miRNAs in the mammalian system with respect to controlling cell proliferation, lineage determination, differentiation, apoptosis, etc [11], [12]. Dysregulation of miRNAs has been associated with a number of disease states including cancers, cardiomyopathies, neurological disorders and deafness [13]–[17]. However, there are only a few reports of genetic mutations leading to skeletal defects in humans. One study showed that germline deletion of the miR-17∼92 cluster causes human skeletal and growth defects involving microcephaly, short stature and digital abnormalities [18]. A mutation in the 3′UTR of the human *HDAC6* gene, which disrupts a miR-433 binding site, was reported to cause a dominant X-linked chondrodysplasia [19]. Another report revealed a link between human juvenile osteoporosis and a mutation in the precursor sequence of miR-2861 [20]. It is predicted that many more mutations will be reported as we continue to learn about the functions of miRNAs in skeletal biology [21]–[23].

To date, there is limited information on the expression and function of specific miRNAs in cartilage development *in vivo*. What is known, however, has predominantly come from studies in mice. Conditional knock-out of the pre-miRNA processing enzyme, *Dicer*, was shown to cause severe skeletal growth defects due to alterations in chondrocyte proliferation and hypertrophy [24]. To date, miR-140 is the best-described miRNA in cartilage since it was identified as abundantly expressed and almost specific to cartilaginous tissues during zebrafish and mouse development [25], [26]. miR-140 null mice have been generated by two independent groups that reported craniofacial deformities and dwarfism due to defects in growth plate cartilage of long bones [27], [28]. A role for miR-140 in regulating homeostasis of mature articular cartilage has also been proposed [27]. In addition to studies on miR-140, there is an increasing number of reports on expression and function of other miRNAs *in vitro*. Among such studies, attempts have been made to define miRNA expression patterns and function during *in vitro* chondrogenesis of primary stem cells or precursor cell lines [29]–[31]. Approaches have also been applied to study miRNA expression in primary chondrocytes, comparing differentiated vs de-differentiated cells or normal vs osteoarthritic chondrocytes, for example [31]–[39]. While these studies are important, they have not provided information on the role of miRNAs in regulating specific phases of chondrocyte differentiation *in vivo* during growth plate or articular cartilage development, for example. To attempt to bridge this knowledge gap, we investigated differential miRNA expression patterns within human embryonic cartilage tissue. Specifically, we chose a time point of development prior to endochondral bone formation (gestational day 54–56) where three populations of chondrocytes can be distinguished in tissue sections of the human embryonic limb. Utilizing laser capture microdissection, cartilage tissue containing either precursor chondrocytes, differentiated chondrocytes or hypertrophic chondrocytes was isolated and expression of miRNAs in each region was determined by TaqMan® OpenArray® analysis. Here, we report on those miRNAs that were found to be significantly differentially expressed between chondrocytes *in vivo* at these three specific stages of development. Data acquired from this study will be an important resource of information toward a better understanding of miRNA function in regulating cartilage and long bone development and disease. Additionally, findings from this research may aid toward the development of future miR-based tissue engineering strategies to promote articular cartilage or endochondral bone repair or regeneration.

## Materials and Methods

### Tissue source and ethics statement

Human, normal embryonic tissue samples (limbs) at gestational day 54–56 were obtained from a tissue collection and distribution program at the Laboratory of Developmental Biology (LDB) within the Department of Pediatrics and Medicine at the University of Washington in Seattle. This service provides precisely-staged normal human embryonic tissue specimens to grant-funded researchers nationally and internationally [40]–[42]. Activities of this Laboratory are IRB-approved by the University of Washington Human Subjects Division (protocol # 41557). These approved activities include the documentation of written informed consent by the donor participants to collect tissue following surgery and to distribute the tissue to researchers. Funding for this tissue collection and distribution service is currently provided from the National Institute of Child Health and Human Development of the National Institutes of Health (R24 HD000836). Request to work with this human embryonic tissue was reviewed by the Human Research Protection Office (HRPO) at Washington University in St Louis. This project was deemed exempt since it did not constitute human subjects research. This was due to the fact that receiving embryonic tissue from University of Washington would not involve obtaining data through intervention or interaction with a living individual. Also, other than gestational age, no identifying information was provided upon receipt of the tissue. Limb tissues obtained by LDB Staff were frozen immediately in coronal orientation in Tissue-Tek® O.C.T. compound and shipped overnight to the McAlinden Laboratory. Tissue was stored at minus 80°C for no longer than 2 wk before collecting frozen sections. In some cases, tissue collected by LDB Staff was immediately fixed in 10% formalin and shipped to the McAlinden Laboratory within 24 h. Upon receipt, tissue was processed immediately for paraffin embedding.

### Histology and immunofluorescence

Paraffin sections were treated with xylene, rehydrated through decreasing concentrations of ethanol, stained in Weigert's Hematoxylin for 5 min, washed in running water for 5 min and stained with 0.001% Fast Green for 3 min. Samples were then rinsed in 1% glacial acetic acid and stained in 0.1% Safranin O for 5 min. Samples were dehydrated and cleared by incubation in 95% alcohol, 100% alcohol and then xylene. For immunofluorescent antibody (Ab) staining, de-paraffinized sections were treated with 1% hyaluronidase (Sigma) for 30 min at 37°C. Sections were rinsed with 1× PBS and blocked with 10% goat serum for 1 h at room temperature and then incubated overnight at 4°C with the following primary antibodies: 1) a rabbit polyclonal “anti-IIA” antibody (Ab) that recognizes the exon 2-encoded cysteine-rich domain of the amino propeptide of type II procollagen (1/400 dilution) [43]; 2) a rat polyclonal Ab against the triple helical domain of type II collagen (1/100 dilution) [44]; 3) a rabbit polyclonal Ab against type I collagen (1/300 dilution) (abcam®); and 4) a rabbit polyclonal Ab against the hypertrophic chondrocyte marker, type X collagen (1/1000 dilution) [45], [46]. Each antibody was diluted in 2% goat serum. Following 1× PBS washes, paraffin sections were incubated with species-specific secondary antibodies (1/250 dilution) that were conjugated to Alexa fluorescent dyes (Invitrogen: goat anti-rabbit Alexa 488; goat anti-rat Alexa 594) for 1 h at room temperature. DAPI mounting medium was applied following three rinses in 1× PBS and stained sections were cover-slipped. A Nikon Eclipse E800 fluorescence microscope was used to view the fluorescent images. The FITC and TRITC band pass filter sets were used to view sections labeled with Alexa 488 and 594 dyes, respectively and the DAPI filter set was used for viewing cell nuclei.

### Laser capture microdissection and RNA isolation

Frozen blocks of human embryonic limb tissue were sectioned (20 µm) onto RNAse-free polyethylene naphthalate (PEN)-coated glass slides (Leica Microsystems) that are specifically designed for laser capture microdissection (LCM). Frozen sections were stored at minus 80°C for up to 48 h before being processed for LCM. Upon removal from cold storage, the tissue sections were exposed to 30 s incubations in cold 75% ethanol followed by 50% ethanol, 20% ethanol, dH_2_O, 0.02% Toluidine Blue (prepared in RNAse free H_2_O), two dH_2_0 washes, 75% ethanol, 95% ethanol and 100% ethanol. Finally, sections were transferred to xylene for 3 min and air-dried. LCM was immediately carried out using the LMD7000 system (Leica Microsystems). Three cartilage regions of interest in each tissue section were selected using the system software resulting in a contact and contamination-free collection of tissue directly into one of the three sterile 0.5 mL Eppendorf caps containing 60 µl of lysis buffer from the RNA isolation kit of choice. This procedure was repeated for all tissue sections derived from one limb specimen (generally 35–40 sections per limb) and laser-captured tissue was pooled together into the appropriate collection vial. In some cases, both limbs were obtained from the same embryo and tissue collected from sections of each limb was pooled and counted as one specimen. Subsequently, for each specimen (whether one or two limbs were received), three separate Eppendorf tubes containing tissue from the precursor chondrocyte (PC), differentiated chondrocyte (DC) or hypertrophic chondrocyte (HYP) regions were obtained. Note than PC, DC or HYP tissue was dissected from both the developing femur and tibia and pooled together. A total of nine independent specimens were processed for LCM. Total RNA, including small miRNAs, was isolated from laser capture microdissected tissue using either the Total RNA Purification Micro Kit (Norgen Biotek Corp.) or the *mir*Vana™ miRNA Isolation Kit (Ambion®). Both assay kits were comparable with respect to the RNA yield (ranging from 15–100 ng/µL depending on the number of tissue sections pooled) as measured by spectrophotometry (NanoDrop™; Thermo Scientific).

### TaqMan® OpenArray® technology to determine microRNA expression profiles

The TaqMan® OpenArray® Human MicroRNA Panel enables simultaneous running of hundreds of TaqMan® MicroRNA Assays in a plate format on the Applied Biosystems OpenArray® Real-Time PCR system [47]. To prepare samples for OpenArray® analysis, mature miRNAs in the RNA samples obtained by LCM were reverse transcribed using Megaplex™ RT primers in a set of two pre-defined pools (Pool A and Pool B), each pool containing up to 381 stem-looped RT primers. Depending on the concentration of RNA obtained from each specimen, 30 ng of RNA was used for the RT step (lower than the recommended amount of 100 ng). The recommended RT thermal cycling conditions were used: 16°C, 2 min; [42°C, 1 min; 50°C, 1 sec for 40 cycles]; 85°C, 5 min; 4°C hold. An aliquot of the RT reaction (5 µL instead of the recommended 2.5 µL) was used for the non-biased pre-amplification step to increase the quantity of cDNA prior to PCR on the TaqMan® OpenArray® MicroRNA Panel. PreAmp Reaction Mix, containing PreAmp Primer Pool A or Pool B and TaqMan® PreAmp Master Mix (20 µL) was added to RT cDNA (5 µL) and the final 25 µL reaction mix underwent pre-amplification using the following thermal cycling parameters: 95°C 10 min; 55°C, 2 min; 72°C, 2 min; [95°C, 15 sec, 60°C, 4 min for 15 cycles], 99.9°C, 10 min; 4°C hold. The number of cycles in this pre-amplification step was increased from the recommended 12 cycles to 15 cycles. The pre-amplification products (5 µL) were diluted in 0.1X TE buffer (95 µL) before adding the samples on the TaqMan® OpenArray® MicroPanels (a 20 fold dilution instead of the recommended 40 fold dilution of pre-amplified cDNA products). Finally, trained staff at Washington University Genome Technology Access Center loaded the diluted pre-amplification samples onto the TaqMan® OpenArray® MicroRNA Panels using the AccuFill™ System and PCR was carried out on the OpenArray® Real-Time PCR System using the manufacturer's instructions. Note that the alterations in the recommended protocol (i.e. reduced levels of RNA for the RT step, increased pre-amplification cycle numbers and reduced dilution of the pre-amplified cDNA samples) were made due to the relatively low concentrations of RNA obtained from laser capture microdissection. Importantly, technical staff at Life Technologies (Dr. Yu Liang, personal communication) tested the feasibility of using the adjusted protocol by analyzing human lung RNA samples and comparing miRNA expression data generated from the adjusted protocol and the recommended protocol. miRNA expression profiles were found to correlate well between the recommended and adjusted protocols (**Figure S1**).

### Analysis of TaqMan® OpenArray® microRNA expression data

Expression data were processed using OpenArray® Real-Time qPCR Analysis Software (BioTrove™, version 1.0.4). This software processed raw fluorescent signal and generated the cycle threshold (Ct) and cycle threshold confidence value for each assay within the array. A Ct confidence value threshold of 150 was used to identify assays with a reliable Ct value. After this filtering, Ct values were imported into the DataAssist™ software (Applied Biosystems, version 2.7). The maximum allowable Ct value was set at 29. This Ct cut-off was chosen since OpenArray® reactions are carried out in small volumes (33 nL); a single molecule is more concentrated in a smaller reaction volume and amplifies sooner than it would from regular microplate qPCRs. Personal communication with Life Technologies technicians informed us that single copy numbers will produce, on average, a Ct value of 29 with TaqMan® assays. The correlation of Ct values between samples from the same cartilage region was examined to identify any samples with potential low data quality. For normalization using an endogenous control, the stability scores chart was used to identify which one of the three endogenous control RNAs (RNU44, RNU48 or U6 rRNA) had the most stable expression in multiple samples. For global normalization, the average of Ct values for all the assays excluding the three endogenous controls was calculated and this value was used as the background setting. The Ct value of the selected background was subtracted from the Ct value of each assay to calculate the ΔCt values. To identify highest-expressed miRNAs, the average normalized expression level of a miRNA from all samples was calculated (average 2^−ΔCt^). The same list of most highly-expressed miRNAs was generated regardless of whether RNU44 or global mean normalization was used. Therefore, data on differentially-expressed miRNAs was calculated using RNU44 normalization. Using the normalized ΔCt values, the fold change between two groups of samples collected from two different cartilage regions was calculated as mean (2^(−ΔCt^_1_^)^)/mean (2^(−ΔCt^_2_^)^). Significance Analysis of Microarrays (SAM) [48]–[51] was used to calculate the significance of differential expression. miRNAs with at least a two fold change in expression between cells of two cartilage regions, at a false discovery rate less than or equal to 5% (q-value), were identified as differentially expressed. Raw data has been submitted to the GEO data depository (online at www.ncbi.nlm.nih.gov/geo/) and assigned the accession code: GSE49152.

### Pathway analysis of potential microRNA target genes

Following identification of miRNAs that are differentially-expressed between the three cartilage regions (PC, DC and HYP), potential target genes of miRNAs within each comparison group were predicted using three algorithms (TargetScan, miRanda, PicTar) [6], [52], [53]. For miRanda target prediction, target genes were selected if they contained at least one binding site with an mirSVR score≤−0.1. For TargetScan prediction, target genes with at least one conserved miRNA binding site were selected. Overall, for each miRNA in question, genes predicted to be targets by at least two of the three algorithms were identified. This was repeated for each miRNA listed in each comparison group: [i) PC>DC; ii) PC<DC; iii) DC>HYP and iv) DC<HYP] and all potential target genes were consolidated and entered into MetaCore™ (Thomson Reuters Systems Biology Solutions) to characterize biological pathways involving these potential target genes. Pathway maps were used in enrichment analysis and significant pathways were identified using a hypergeometric p-value cut-off of 0.05. This approach has been used in previous studies to identify pathways underlying predicted targets of miRNAs [54], [55]. We did not include differentially expressed miRNAs found in the PC vs HYP comparisons for these analyses since many of the same miRNAs listed here were also found in the other comparisons. Also PC and HYP cells are at extreme ends of the differentiation spectrum. At this stage, we were initially interested in identifying enriched pathways that may be regulated by miRNAs through the normal course of differentiation (i.e. PC →DC → HYP cells).

## Results

### Tissue selection for laser capture microdissection

**Figure 1:**
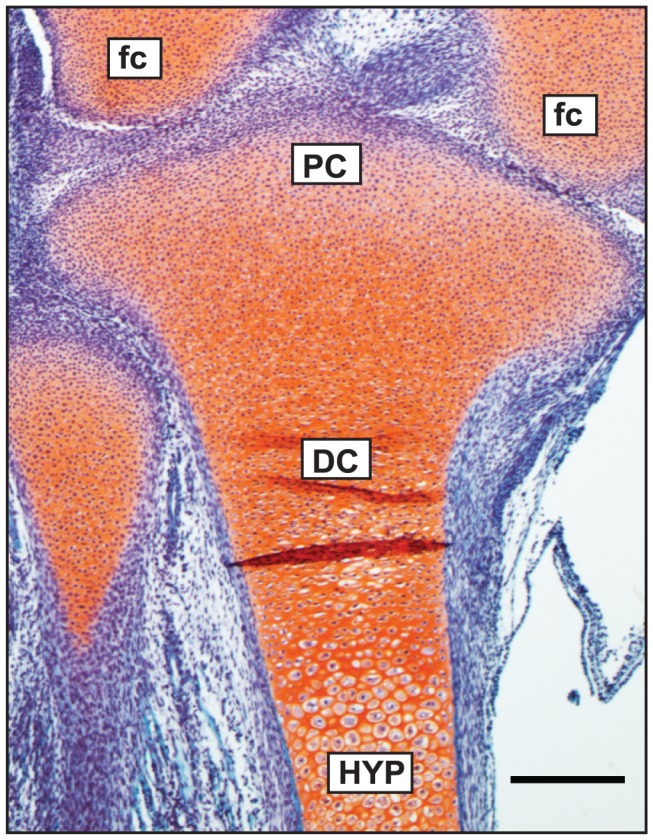
Safranin-O-stained tissue section of a human embryonic developing proximal tibia (gestational day 54). Red-orange staining represents proteoglycans in the developing cartilage extracellular matrix. This stage of development is prior to endochondral ossification. Chondrocytes at various stages of differentiation are present at this stage: precursor chondrocytes (**PC**) are found at the most proximal end of the developing tibia as well as in the surrounding perichondrium; differentiated chondrocytes (**DC**) are located further down the developing limb and are distinguishable by their cuboidal or flattened phenotype, depending on their location; hypertrophic chondrocytes (**HYP**) are terminally-differentiated cells easily distinguished by their increased size. Also shown in this image are the developing femoral condyles (fc) of the distal femur. Scale bar = 250 µm.

**Figure 2:**
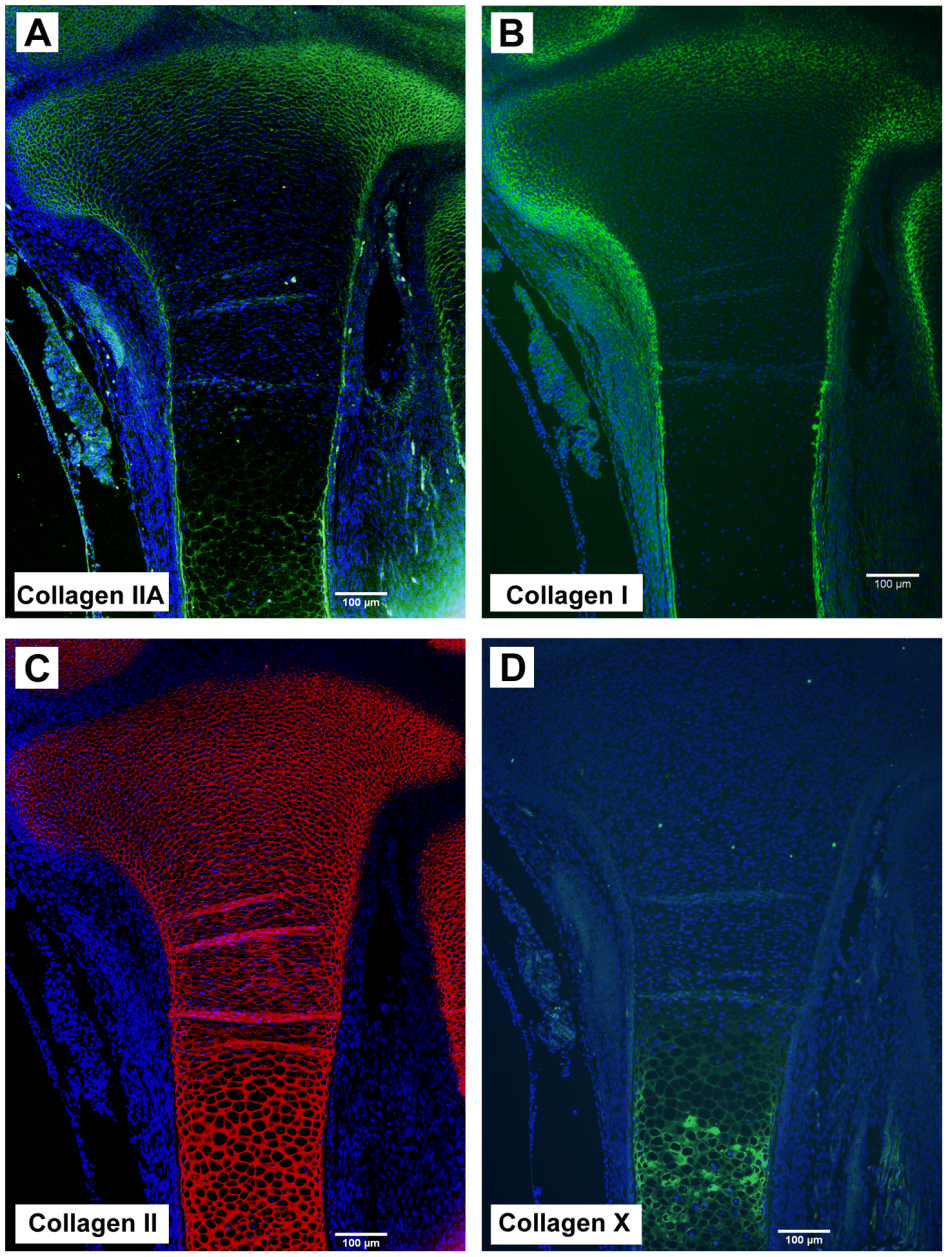
Immunofluorescence staining of different collagen types in a human developing embryonic proximal tibia (gestational day 54). (**A**) Localization of the embryonic isoform of type II procollagen (type IIA) in the extracellular matrix (ECM). The anti-IIA antibody recognizes the exon 2-encoded cysteine-rich domain present in the amino propeptide of type IIA procollagen [43]. These IIA isoforms are generated predominantly by progenitor chondrocytes seen at the periphery and most proximal area of the developing tibia. Some expression of IIA procollagen has been reported in the pre-hypertrophic and hypertrophic region of developing cartilage [58] and is also shown here. (**B**) Type I collagen staining is restricted to the areas of precursor chondrocytes and cells of the perichondrium/periosteum. (**C**) Type II collagen staining patterns (i.e. the processed triple helical domain of type II collagen) is present throughout the entire developing limb. (**D**) Collagen X staining is restricted to the ECM containing hypertrophic chondrocytes. Cell nuclei are visualized in blue by DAPI staining. Scale bars = 100 µm. Immunofluorescent images are representative of three independent experiments using gestational day 54 tissue sections from different embryos.

Human embryonic limbs at day 54–56 of gestational development were chosen for these studies to separate three specific regions containing chondrocytes at distinct stages of differentiation. **Figure 1** shows a proteoglycan-stained (Safranin O) tissue section of a gestational day 54 human limb focusing in on the developing proximal tibia. Three regions are denoted that predominantly contain: 1) precursor chondrocytes (PC) at the most proximal end and at the perimeter within the surrounding perichondrium; 2) differentiated chondrocytes (DC) within the mid-region of the developing limb, some of which show an obvious flattened phenotype; 3) enlarged hypertrophic chondrocytes (HYP) that will eventually undergo programmed cell death by apoptosis. This stage of development is just prior to vascular invasion of the hypertrophic cartilage and subsequent endochondral ossification. Collagen immunolocalization confirms the location of the PC region by positive staining of the “embryonic” IIA procollagen isoform (**Figure 2A**) and type I collagen (**Figure 2B**), two known markers of chondroprogenitor cells. The “IIA” antibody recognizes the conserved cysteine-rich protein domain encoded by alternatively-spliced exon 2 of the *COL2A1* gene [43]. Exon 2 is present in mRNA generated by chondroprogenitor cells, whereas differentiated chondrocytes generate mRNA devoid of exon 2 (encoding type IIB procollagen) [56], [57]. Chondrocytes in a more differentiated state do not express type IIA procollagen or type I collagen. The presence of some IIA protein staining in the hypertrophic zone (**Figure 2A**) has also been reported before in human limb tissue at a similar time-point of development [58]. Type II collagen (i.e. the triple-helical domain of processed type II procollagen) is present throughout the entire developing limb, as expected (**Figure 2C**). Therefore, the DC region was selected based on both cell phenotype (as seen by histology; **Figure 1**) and by the presence of processed type II collagen and the absence of type IIA and type I collagens (**Figures 2A, B**). Type X collagen is a marker of hypertrophic chondrocytes and is localized to the HYP region of the developing limb (**Figure 2D**).

### MicroRNA expression analysis

**Figure 3:**
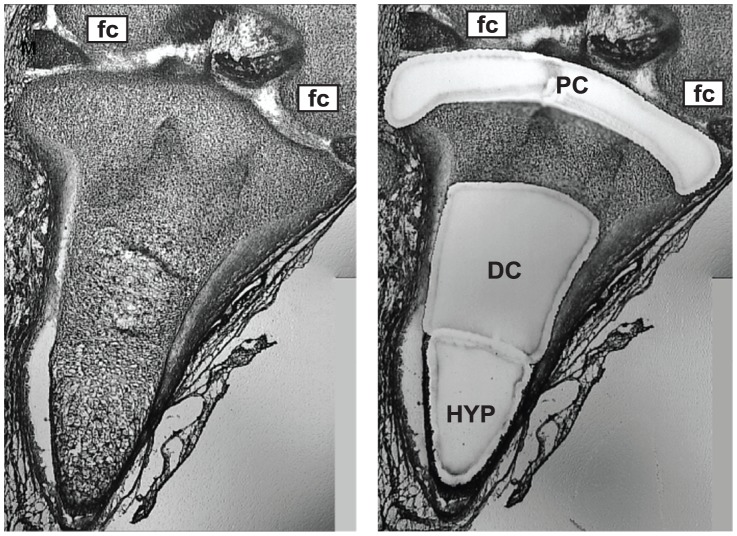
Laser capture microdissection of a human embryonic tibia tissue section (gestational day 54). Left panel shows a processed frozen tissue section and the right panel shows the same tissue section following laser capture microdissection to separate regions containing precursor chondrocytes (PC), differentiated chondrocytes (DC) or hypertrophic chondrocytes (HYP). Also in view is cartilage tissue of the femoral condyles (fc); the developing femur was also micro-dissected to separate the three regions of cartilage. Images were taken using the LMD7000 laser capture microscope set at the 6× objective.

Laser capture microdissection of tissue sections from nine independent limb specimens was successfully carried out as shown in **Figure 3**. Note than an area between the PC and DC region was not isolated by laser capture microdissection. It is expected that this “grey area” contains a mixture of progenitor and differentiated chondrocytes; inclusion of cells from this site would likely dilute the data resulting in lower numbers of differentially-expressed miRNAs identified between PC and DC regions. RNA extracted from PC, DC and HYP areas was processed for analysis on TaqMan® OpenArray® Human MicroRNA Panels and expression of over 750 miRNAs was determined. One “PC” sample was discarded from further analysis for its low data correlation with other PC samples. **Table 1** lists the top thirty most abundantly expressed miRNAs in chondrocytes from PC, DC and HYP regions. Notably, miR-140-5p, the best-described miRNA in cartilage to date, was found to be one of the most highly expressed (albeit not differentially-expressed) miRNAs in all three of the cartilage regions analyzed, thus providing additional confidence in the array data.

**Table 1:** Top 30 most abundantly expressed miRNAs in precursor, differentiated and hypertrophic chondrocytes from gestational day 54–56 human embryonic cartilage tissue.

Precursor Chondrocytes (PC)		Differentiated Chondrocytes (DC)		Hypertrophic Chondrocytes (HYP)	
**miRNA**	**Average(2^−ΔCt^)**	**miRNA**	**Average(2^−ΔCt^)**	**miRNA**	**Average(2^−ΔCt^)**
miR-140-5p	10.76	miR-140-5p	10.72	miR-24	6.66
miR-125b	6.84	miR-24	7.73	miR-92a	5.88
miR-19b	5.82	miR-19b	4.04	miR-140-5p	5.82
miR-30c	4.78	miR-376a	3.66	miR-19b	5.07
miR-199a-3p	4.39	miR-125b	3.38	miR-20a	4.08
miR-92a	3.69	miR-92a	3.25	miR-106a	3.40
miR-376a	3.25	miR-127	2.56	miR-127	3.06
miR-214	2.99	miR-409-3p	2.23	miR-193b	2.92
miR-26a	2.61	miR-30c	2.21	miR-212	2.85
miR-99b	2.53	miR-193b	2.09	miR-30c	2.54
miR-30b	2.40	miR-30b	1.94	miR-17	2.52
miR-382	2.37	miR-26a	1.87	miR-376a	2.33
miR-409-3p	2.21	miR-199a-3p	1.87	miR-125b	2.11
miR-106a	2.02	miR-20a	1.85	miR-30b	1.81
miR-130a	2.02	miR-214	1.67	miR-409-3p	1.60
miR-20a	1.75	miR-574-3p	1.66	miR-26a	1.40
miR-484	1.56	miR-106a	1.62	miR-214	1.39
miR-27b	1.46	miR-99b	1.36	miR-199a-3p	1.31
miR-127	1.45	miR-130a	1.29	miR-484	1.28
miR-331	1.39	miR-484	1.25	miR-181a-2	1.26
miR-206	1.36	miR-212	1.23	miR-206	1.15
miR-574-3p	1.33	miR-455-3p	1.19	miR-210	1.11
miR-455-3p	1.19	miR-206	1.05	miR-99b	1.06
miR-26b	1.17	miR-331	1.01	miR-331	1.06
miR-335	1.12	miR-370	1.00	miR-574-3p	1.00
miR-320	1.12	miR-210	0.82	miR-455-3p	0.90
miR-100	1.12	miR-17	0.80	miR-191	0.88
miR-23b	1.10	miR-27b	0.78	miR-133a	0.76
miR-210	0.97	miR-191	0.78	miR-320	0.72
miR-191	0.96	miR-410	0.73	miR-370	0.69

Highly expressed miRNAs were identified according to their average (2^-ΔCt^) values. Delta (Δ) Ct value for each miRNA was calculated by subtracting the Ct value of endogenous control, RNU44, from the Ct value of the specific miRNA. Expression level average (2^−ΔCt^) in a region reflects the average of 2^−ΔCt^ values across all samples in that region.

### Differentially-expressed microRNAs

From the OpenArray® expression data, we identified miRNAs that were differentially expressed by 2 fold or more between: 1) PC and DC (**Table 2**); 2) PC and HYP (**Table 3**) and 3) DC and HYP regions (**Table 4**). It is noteworthy that the number of differentially-expressed miRNAs was greatest when comparing cells between the PC and HYP regions (**Table 3**). This is not surprising given the fact that precursor and hypertrophic chondrocytes are at extreme ends of the differentiation spectrum. Also, we did not identify any miRNAs that were exclusively expressed in one region but absent from another region of human embryonic developing cartilage. In many cases, the same miRNAs were found to be differentially-expressed in more than one comparison. Fewer miRNAs were identified as being significantly more highly-expressed in DC or HYP chondrocytes when compared to PC cells. For example, miRs-138, 193b and 365 were the only ones found to be expressed at higher levels in DC compared to PC cells (**Table 2**). These miRNAs were also more highly expressed in HYP cells when compared to PC cells, suggesting function(s) in regulating gene pathways associated with later stages of chondrogenesis. Those miRNAs that were specifically more abundantly-expressed in HYP cells compared to DC or PC cells suggests functional role(s) in regulating terminal differentiation of chondrocytes and/or endochondral ossification processes (**Tables 3** **and** **4**). As will be elaborated on in the Discussion, a number of differentially-expressed miRNAs identified in this study have been described in other aspects of cartilage biology and in other systems that may provide clues toward identifying their function in regulating specific stages of chondrocyte differentiation. Importantly, this study has identified a number of miRNAs that have not yet been reported to be expressed in cartilage chondrocytes.

**Table 2:** Differentially-expressed miRNAs between precursor chondrocytes (PC) and differentiated chondrocytes (DC) from gestational day 54–56 human embryonic cartilage tissue.

PC compared to DC							
PC>DC				PC<DC			
**miRNA**	**f.c.**	**Score**	**q-value**	**miRNA**	**f.c.**	**Score**	**q-value**
miR-224	5.09	3.59	0	miR-138	-12.18	-3.29	2.09
miR-1247	4.59	4.21	0	miR-193b	-3.63	-4.63	0
miR-335*	4.37	3.16	0	miR-365	-2.45	-3.45	0
miR-532	4.25	3.67	0				
miR-146b	3.55	3.97	0				
miR-24-2-5p	3.33	2.17	2.09				
miR-660	3.32	3.17	0				
miR-502-3p	3.23	2.68	0				
miR-532-3p	3.16	3.61	0				
miR-27a	2.99	2.86	0				
miR-10b-3p	2.83	3.84	0				
miR-708	2.75	1.87	3.16				
miR-100	2.58	2.90	0				
miR-29c	2.57	2.87	0				
miR-199a-3p	2.35	2.95	0				
miR-196b	2.30	3.22	0				
miR-30d	2.18	1.90	3.16				
miR-30c	2.16	2.24	2.09				
miR-454	2.12	3.16	0				
miR-151-5p	2.09	1.90	3.16				
miR-323-3p	2.09	3.25	0				
miR-335	2.09	2.31	0				
miR-99b*	2.05	2.65	0				

Fold change (f.c.) expression of miRNAs between cells of PC and DC regions are shown. The score (d) and q-values for each differentially-expressed miRNA are shown based on SAM analysis (FDR≤5%; n = 8–9).

**Table 3:** Differentially-expressed miRNAs between precursor chondrocytes (PC) and hypertrophic chondrocytes (HYP) from gestational day 54–56 human embryonic cartilage tissue.

PC compared to HYP							
PC>HYP				PC<HYP			
**miRNA**	**f.c.**	**Score**	**q-value**	**miRNA**	**f.c.**	**Score**	**q-value**
miR-335*	40.38	3.85	0	miR-181a-1	-17.36	-3.22	0.54
miR-532-5p	10.85	4.30	0	miR-138	-16.61	-3.50	0
miR-224	10.22	3.46	0	miR-193b	-5.06	-6.24	0
miR-660	8.62	4.02	0	miR-150	-4.33	-2.07	4.42
miR-483-3p	8.19	12.88	0	miR-1291	-3.88	-2.41	3.92
miR-335	7.92	4.20	0	miR-193b*	-3.75	-2.10	4.42
miR-532-3p	7.17	4.67	0	miR-181a-2	-3.26	-2.36	4.42
miR-146b	6.87	4.88	0	miR-1290	-2.98	-2.27	4.42
miR-502-3p	6.41	2.62	0	miR-202	-2.91	-1.99	4.42
miR-196b	5.30	5.20	0	miR-17	-2.75	-3.99	0
miR-125a-5p	5.24	3.21	0	miR-365	-2.74	-2.30	4.42
miR-93	4.65	1.76	0.94	miR-222	-2.70	-2.77	2.63
miR-301	4.26	3.20	0	miR-139-5p	-2.57	-3.16	0.54
miR-323-3p	4.07	5.23	0	miR-20a	-2.33	-2.73	2.63
miR-10b-3p	4.03	5.18	0	miR-126	-2.22	-1.96	4.42
miR-100	3.97	4.06	0	miR-127	-2.10	-2.56	3.92
miR-454	3.87	4.82	0				
miR-151-5p	3.80	2.54	0				
miR-16	3.65	4.00	0				
miR-758	3.49	2.18	0.54				
miR-93*	3.45	4.32	0				
miR-199a-3p	3.36	3.80	0				
miR-320b	3.34	3.24	0				
miR-130a	3.28	2.78	0				
miR-125b	3.24	2.22	0				
miR-708	3.20	2.14	0.54				
miR-25	3.13	2.82	0				
miR-495	3.03	3.46	0				
miR-675	2.98	2.95	0				
miR-27b	2.96	3.11	0				
miR-337-3p	2.86	1.96	0.54				
miR-24-2-5p	2.83	1.47	2.63				
miR-939	2.81	1.23	3.92				
miR-199b	2.80	1.86	0.95				
miR-30d	2.72	2.28	0				
miR-543	2.68	2.94	0				
miR-889	2.67	2.21	0.54				
miR-29c	2.60	2.78	0				
miR-148a	2.52	2.37	0				
miR-26b	2.51	2.34	0				
miR-1180	2.42	2.64	0				
miR-296	2.41	2.12	0.54				
miR-99b	2.38	4.19	0				
miR-27a	2.32	2.55	0				
miR-625*	2.32	1.56	2.63				
miR-199a	2.28	1.89	0.95				
miR-369-3p	2.25	1.20	3.92				
miR-380-5p	2.24	1.51	2.63				
miR-1244	2.20	1.39	2.63				
miR-214	2.15	2.61	0				
miR-433	2.14	2.61	0				
miR-99b*	2.14	2.69	0				
miR-301b	2.11	1.86	0.95				
miR-106b	2.09	2.12	0.54				
miR-769-5p	2.00	2.72	0				

Fold change (f.c.) expression of miRNAs between cells of PC and HYP regions are shown. The score (d) and q-values for each differentially-expressed miRNA are shown based on SAM analysis (FDR≤5%; n = 8–9).

**Table 4:** Differentially-expressed miRNAs between differentiated chondrocytes (DC) and hypertrophic chondrocytes (HYP) from gestational day 54–56 human embryonic cartilage tissue.

DC compared to HYP							
DC>HYP				DC<HYP			
**miRNA**	**f.c.**	**Score**	**q-value**	**miRNA**	**f.c.**	**Score**	**q-value**
miR-335*	9.23	7.97	0	miR-181a-1	-6.17	-3.16	0
miR-483-3p	4.53	4.55	0	miR-181a-2	-3.79	-3.27	0
miR-335	3.79	3.96	0	miR-17	-3.16	-4.07	0
miR-301	2.94	2.86	0	miR-1290	-2.72	-2.44	4.39
miR-320b	2.74	1.64	4.39	miR-20a	-2.20	-2.47	4.39
miR-758	2.72	3.08	0	miR-1260	-2.01	-2.48	4.39
miR-125a-5p	2.71	3.25	0				
miR-16	2.69	2.57	0				
miR-380-5p	2.60	3.34	0				
miR-660	2.59	2.65	0				
miR-532-5p	2.55	1.80	2.41				
miR-196b	2.30	2.75	0				
miR-532-3p	2.26	3.54	0				
miR-130a	2.11	1.89	2.41				
miR-184	2.06	2.05	2.41				
miR-542-3p	2.06	1.99	2.41				
miR-93*	2.05	3.60	0				
miR-1244	2.03	2.02	2.41				
miR-224	2.00	2.18	2.41				
miR-495	2.00	3.31	0				

Fold change (f.c.) expression of miRNAs between cells of DC and HYP regions are shown. The score (d) and q-values for each differentially-expressed miRNA are shown based on SAM analysis (FDR≤5%; n = 8–9).

### Pathway analysis of predicted microRNA target genes

Potential target genes were identified for each miRNA listed in **Tables 2** **and** **4** using defined criteria for target selection (i.e. targets predicted by at least two of three chosen algorithms; see Materials and Methods). All predicted target genes for miRNAs differentially expressed between cells in the PC and DC region and those differentially-expressed between cells of the DC and HYP regions were entered into MetaCore™ to identify cellular pathways that are underlying these genes. Many significant “over-represented” pathways were identified for each comparison [i) PC>DC; ii) PC<DC; iii) DC>HYP; iv) DC<HYP] including those involved in cytoskeletal remodeling, cell cycle, transcription, apoptosis etc. At this stage, we have focused only on developmental pathways that have relevance within the context of chondrogenesis. **Tables 5** **,** **6** **,** **7** **,** **8** list the top 6–7 developmental pathways that may be regulated by miRNA groups that we have shown are more highly expressed within a specific region of developing cartilage tissue. Enriched pathways common between all comparisons include those related to growth factor signaling/regulation that are known to be important at various stages of chondrocyte differentiation (i.e. IGF-1, TGF-β, BMP, FGF signaling pathways; **Tables 5** **,** **6** **,** **7** **,** **8**). Interestingly, the majority of enriched pathways of predicted genes targeted by miRNAs more highly expressed in DC compared to PC cells were related to Hedgehog and Wnt signaling pathways (**Table 6**). VEGF signaling pathways were implicated to be regulated by miRNAs more highly expressed in PC cells (**Table 5**) or DC cells (**Table 7**). Notably, the developmental pathway involving IL-8 in angiogenesis was specific to the group of miRNAs more highly expressed in HYP cells compared to DC cells (**Table 8**). For a list of the genes involved in each significantly-enriched pathway that were found to be potential targets of miRNAs in each differentially-expressed group, **see Tables S1, S2, S3, S4**.

**Table 5:** Enriched pathways of predicted genes targeted by differentially expressed miRNAs (PC>DC).

**Developmental Pathways**	**p-value**
IGF-1 receptor signaling	4.364E-11
TGF-β receptor signaling	4.495E-08
BMP signaling	5.546E-07
FGF receptor signaling	9.964E-07
Role of activin A in cell differentiation and proliferation	8.405E-06
FGF family signaling	5.546E-05
VEGF family signaling	5.844E-05

**Table 6:** Enriched pathways of predicted genes targeted by differentially expressed miRNAs (PC<DC).

Developmental Pathways	p-value
IGF-1 receptor signaling	1.783E-05
Hedgehog signaling	5.214E-04
WNT5a signaling	5.867E-04
WNT signaling (general)	1.122E-03
Hedgehog and PTH signaling in bone and cartilage development	1.152E-03
Role of activin A in cell differentiation and proliferation	1.192E-02

**Table 7:** Enriched pathways of predicted genes targeted by differentially expressed miRNAs (DC>HYP).

Developmental Pathways	p-value
TGF-β receptor signaling	4.671E-10
WNT5a signaling	9.408E-09
FGF receptor signaling	1.008E-07
BMP signaling	1.660E-07
Role of activin A in cell differentiation and proliferation	3.126E-07
IGF-1 receptor signaling	2.002E-06
VEGF signaling and activation	5.438E-06

**Table 8:** Enriched pathways of predicted genes targeted by differentially expressed miRNAs (DC<HYP).

Developmental Pathways	p-value
TGF-β receptor signaling	1.252E-07
FGF receptor signaling	3.583E-07
WNT5a signaling	2.962E-06
IGF-1 receptor signaling	9.252E-06
Role of IL-8 in angiogenesis	2.076E-05
Role of activin A in cell differentiation and proliferation	2.452E-05
Hedgehog signaling	8.933E-05

## Discussion

This novel study is the first to report on the *in vivo* expression patterns of miRNAs in chondrocytes from human embryonic cartilage tissue in the developing limb. The significance of this work is further highlighted by the fact that three distinct regions of the developing limb were separated by laser capture microdissection to permit analysis of miRNA expression in precursor chondrocytes (PC), differentiated chondrocytes (DC) or hypertrophic chondrocytes (HYP) of the femur and tibia. While other studies have been carried out to identify mRNA or miRNA expression in chondrocytes from different sites of mouse or chicken cartilage tissue, either late-stage embryonic, neonatal or post-natal tissue was utilized [24], [59], [60]. The type of study described in this report to determine miRNA expression in three distinct populations of chondrocytes at an early stage of human embryonic development (day 54–56 of gestation; prior to primary endochondral ossification) would be extremely challenging in mouse, rat or chicken tissue due to size constraints. Given the many advantages of using human embryonic tissue, it was not possible to confidently delineate and isolate the small region containing pre-hypertrophic chondrocytes in our system. We therefore expect that a proportion of these cells were present in both the DC and HYP samples following laser capture microdissection. Also, it is unclear at present if data generated from this study will be useful toward understanding miRNA-regulated processes that form chondrocytes found in permanent articular cartilage since the exact location/subset of precursor cells that form this tissue is not entirely understood [61], [62] and the time point of development chosen for these studies may not be appropriate in this context. Regardless of these issues, we have generated an important database to begin to decipher miRNAs that may play a functional role in regulating not only chondrogenesis, but also specific phases of chondrocyte differentiation during limb development.

### Highly expressed microRNAs

The miRNA expression database was obtained using Human MicroRNA OpenArray® Panels on the OpenArray® Real-Time PCR system [47]. Since this system determined miRNA expression based on TaqMan® technology, further validation of expression (as is required for hybridization-based microarrays) was not necessary. From **Table 1** that lists the top thirty most highly-expressed miRNAs in PC, DC and HYP, miR-140-5p was found to be one of the most abundant regardless of the status of chondrocyte differentiation. This finding provides confidence in the array data since miR-140 is the best-described miRNA in cartilage to date and is known to be highly expressed and almost specific to cartilaginous tissues. Miyaki *et al* reported higher levels of miR-140 expression in proliferating chondrocytes of mature (post-natal day 10) murine growth plates compared to hypertrophic chondrocytes [27]. In our system, we did not detect significant differences in expression of miR-140 between precursor, differentiated proliferating and hypertrophic chondrocytes. This discrepancy may be explained by species difference as well as the stage of growth plate development analyzed (mature murine vs human embryonic growth plates). These findings may also suggest that miR-140 expression patterns change as the cartilage growth plate matures. Also, Miyaki *et al* detected the larger primary precursor form of miR-140 (pri-miR-140) in murine growth plate sections by *in situ* hybridization that includes both 5p and 3p strand; different expression patterns may be obtained by specifically detecting either the mature 5p or 3p strand *in vivo*. We also detected expression of miR-140-3p in all three regions of developing cartilage, albeit at lower levels than miR-140-5p (∼18-22 fold less depending on the cartilage region; results not shown). However, two studies have reported higher expression of the 3p strand in mature rat or neonatal murine epiphyseal growth plate cartilage [24], [63]. This suggests that both the 5p and 3p strands of miR-140 are functional and that their ratio levels could change depending on developmental time point. It will also be interesting to determine expression levels of miR-140-3p and -5p in osteoarthritic cartilage given a recent report describing regulation of miR-140-3p by the inflammatory cytokine, TNF-α, in airway smooth muscle cells [64]. Therefore, in addition to a role for miR-140 in regulating development and homeostasis of cartilage tissue [27], [28] additional functions (potentially distinct functions for the 5p and 3p strands) may exist in an inflammatory environment as found in osteoarthritic cartilage.

Other highly-expressed miRNAs include those of the miR-17∼92 cluster (miR-17, miR-20a, miR-19b, miR-92a) (**Table 1**). Interestingly, germline deletion of this cluster has been linked to human skeletal and growth defects [18]. miRs-18a and 19a from this cluster were also expressed in human embryonic chondrocytes, but not as highly as the other miRNAs in the cluster. Also, our studies showed that miR-17 and miR-20a were found to be significantly more highly-expressed in hypertrophic chondrocytes compared to both precursor and differentiated chondrocytes (**Tables 3** **and** **4**). These expression patterns suggest that miRNAs in this cluster may be distinctly regulated and that some of them (i.e. miRs-17 and 20a) may function to regulate terminal hypertrophic chondrocyte differentiation or endochondral ossification. In other systems, the miR-17∼92 cluster has been shown to regulate components of the TGF-β pathway as well as angiogenesis [65]–[70]. Future studies will be required to determine if, in the context of chondrogenesis, miRNAs in this cluster affects TGF-β signaling and endochondral ossification processes. Interestingly, within the top 30 most abundant miRNAs expressed in all regions of developing human cartilage was miR-206, which is known to be a muscle-specific miRNA [71] (**Table 1**). This miRNA was also found to be highly expressed in mouse cartilage [24] and muscle-specific gene expression has been reported recently in murine articular cartilage within the context of ageing and OA [72], [73]. The potential role of these muscle-related miRNAs and genes in cartilage development and disease may be worth pursuing. The miRNA OpenArray® screen also identified high expression of miR-376a in chondrocytes throughout the human developing limb. This miRNA is part of a large cluster, two of which (miR-654/376b) were found to be expressed strongly in murine embryonic cartilage [74]. Our studies also showed that miR-127 and miR-409-3p were strongly expressed at all stages of human embryonic chondrocyte differentiation. Interestingly, these two miRNAs (in addition to miR-376b) were also identified as being more highly expressed in chondrocytes from murine neonatal hind limb cartilage when compared to osteoblasts [24]. We are currently pursuing studies to determine functional roles of these abundantly-expressed miRNAs in the context of cartilage biology.

### Differentially expressed microRNAs

A number of miRNAs identified in this study as significantly differentially expressed between precursor, differentiated and hypertrophic chondrocytes have also been described in other reports within the context of skeletal development or cartilage biology. miR-196b expression patterns indicate a potential role in regulating specific phases of chondrogenesis given that levels were found to be higher in precursor or differentiated chondrocytes (**Tables 2** **,** **3** **,** **4**). In fact, miR-196 sub-types (including both miR-196a and miR-196b, which are almost identical in sequence but located on different chromosomes) have been reported to regulate skeletal patterning in zebrafish, chicken and salamander [75]–[77]. This patterning role is partly explained by the fact that miRs-196a/b are located within *HOX* gene clusters and can regulate expression of some of these patterning genes [76], [78]–[80]. miRs-196a/b have also been shown to regulate *ERG* transcription factors [81]. This is interesting since *ERG* is specifically localized to developing articular cartilage of the joint and functions in regulating proper cartilage development [82]. Increased expression of miR-196a has also been shown to decrease proliferation of adipose-derived stem cells and enhance their osteogenic potential without affecting adipogenesis [83]. It will be important to establish how altered expression of miR-196 affects the chondrogenic potential of precursor stem cells *in vitro*.

Another miRNA identified as over 2-fold more highly expressed in PC compared to HYP is miR-433 (**Table 3**). This miRNA has been linked to chondrodysplasia in humans since a mutation in the 3′UTR of *HDAC6* was identified within a miR-433 binding site [19]. Other differentially-expressed miRNAs found in this study (miRs-27a, 675, 483) (**Tables 2** **,** **3** **,** **4**) have been reported in the context of cartilage and osteoarthritis (OA). miR-27a was found to be expressed in human OA chondrocytes and to indirectly affect expression of IGFBP-5 and MMP-13 [84]. Its differential expression pattern, as seen in our system, suggests a developmental role in controlling early stage chondrocyte differentiation (**Tables 2** **–** **3**). *In vitro* studies showed that miR-675 indirectly affected levels of *COL2A1* in differentiated human articular chondrocytes [32]. Recently Steck *et al* reported that expression of this miRNA was elevated in human OA as was the long non-coding RNA, H19, which harbors miR-675 [85]. Interestingly, H19/miR-675 is located within an imprinted domain on human chromosome 11. H19 is maternally expressed while IGF2 is paternally-expressed and harbors miR-483 within intron 2. Steck *et al* also reported elevated IGF2 levels in OA cartilage while levels of miR-483 were not described in this study. However, other studies have reported increased levels of miR-483-5p in OA cartilage [34], [86]. It will be interesting to further dissect how regulation of chondrocytes by H19/miR-675 and IGF2/miR-483 affects cartilage matrix production and maintenance. In our studies, we detected miR-483-5p expression in human embryonic chondrocytes, but only miR-483-3p was found to be differentially-expressed in precursor and differentiated chondrocytes when compared to hypertrophic chondrocytes (**Tables 3** **and** **4** **)**. Also, expression levels of the 5p and 3p strands of miR-483 in PC and DC regions were similar suggesting that both mature strands are functional in chondrocytes. Whether these 5p and 3p strands have distinct functions in chondrocytes remains to be determined.

Expression of miR-146a has also been reported in OA chondrocytes; mechanistically, miR-146a is apparently responsive to IL-1β signaling and may be involved in pain-related pathophysiology of OA [38], [87], [88]. This miRNA was detected in our screen, but not differentially-expressed between cartilage regions. However, its homologue, miR-146b, was expressed at higher levels than miR-146a and was also found to be more highly-expressed in precursor chondrocytes when compared to differentiated and hypertrophic chondrocytes in our studies (**Tables 2** **and** **3**). miRs-146a and b differ by 2 nucleotides and are located on different chromosomes. Therefore, it is possible that these miRNA homologues are differentially-regulated during chondrogenesis and carry out distinct functions to control developmental processes as well as homeostasis in mature tissue. Studies to address this hypothesis are underway in our laboratory.

Among those miRNAs more highly expressed in differentiated or hypertrophic chondrocytes when compared to precursor chondrocytes, it is interesting that two of these, miRs-365 and 222 (**Tables 2** **and** **3**), have been postulated to play a mechano-regulatory role in cartilage [60], [89]. miR-365 is clustered with miR-193b which shows similar expression patterns to miR-365 in our system (**Tables 2** **and** **3**), thus suggesting that these miRNAs may be co-regulated. Therefore, in addition to a mechano-regulatory role in mature cartilage tissues, our studies suggest a potential developmental role for these miRNAs in regulating later stages of chondrocyte differentiation. Other miRNAs detected in our screen that are more abundantly-expressed in HYP cells compared to cells in the DC and PC regions also suggests functional role(s) in regulating terminal differentiation of chondrocytes and/or endochondral ossification processes (e.g. miRs-181a-1, 181a-2, 17, 1290, 1291, 20a, 1260, 150, 202, 139-5p, 126, 127) (**Tables 3** **and** **4**).

### Other microRNAs not yet reported in cartilage

miR-335-5p and the less abundant 3p strand (miR-335*) showed significantly high fold changes in differential expression patterns in our studies (**Tables 2** **,** **3** **,** **4**). Although expression levels of miR-335* were generally lower than miR-335-5p, detection of this “minor” strand suggests that it may be functional in the context of cartilage biology. While miR-335* has not been reported in cartilage until now, one previous study has shown down-regulation of the miR-335-5p strand in de-differentiated chondrocytes [89]. Unpublished observations in our laboratory have shown a sharp decrease in miR-335-5p expression during TGF-β3 induced chondrocyte differentiation of human MSCs. These findings suggest a potential role for the 5p strand of miR-335 in regulating genes to maintain a more progenitor phenotype. A recent study by Tome *et al* [90] supports this view since they found miR-335-5p down-regulation was required to permit MSC differentiation toward the osteogenic or adipogenic lineage; over-expression of this miRNA inhibited MSC differentiation. However, another study reported opposite effects of miR-335-5p in regulating osteogenesis [91], but this may be explained by the fact that cell lines were used here as opposed to primary MSCs. It will be interesting to determine how modulation of miR-335/335* expression affects chondrocyte differentiation.

This study is the first to report miR-138 in cartilage and that higher expression is associated with differentiated and hypertrophic chondrocytes when compared to precursor cells (∼12 fold and 17 fold difference, respectively; **Tables 2** **and** **3**). These expression patterns potentially indicate a role in regulating specific phases of chondrocyte differentiation. Other studies have shown that over-expression of miR-138 inhibits osteogenic and adipogenic differentiation [92], [93]. Interestingly, it has also been demonstrated that miR-138 can promote induced pluripotent stem cell (iPS) generation via regulation of p53 [94]. This clearly indicates that miR-138 can control cellular differentiation and may function through different mechanisms depending on the tissue microenvironment. It will be important to understand how this miRNA regulates chondrogenesis and the mechanisms involved. In addition to miR-335 and miR-138, there are a number of other differentially-expressed miRNAs identified in the present study that will be worth pursuing in the context of cartilage biology; some of these are generally not well-reported in the literature and their functional roles in normal tissue development and homeostasis are unknown so far (e.g. miRs- 301, 502, 532, 660, 1244, 1247, 1290, 1291).

### Regulation of cellular pathways by microRNAs in cartilage

miRNA function in cartilage development and homeostasis is becoming a rapidly-growing area of research [21]–[23]. This study has identified many miRNAs worth pursuing for their potential function in regulating specific phases of chondrocyte differentiation. However, once functional miRNAs have been identified, the next challenge is to understand the mechanisms by which specific miRNAs, or sub-groups of miRNAs, control cellular processes. While many studies report on a specific miRNA that can interact with and regulate one target gene, this does not reflect the *in vivo* situation since miRNAs can potentially target hundreds of genes (Krek, 2005). Also, the degree of miRNA repression on an individual target is usually very mild [95]–[97] and so to exert significant biological function, miRNAs may regulate multiple genes within the same pathway as has been shown for the miR-16 family of miRNAs and for miR-17-5p [98], [99]. In addition, the target genes and the pathways involved will be different for a specific miRNA or group of miRNAs depending on the tissue type and time point of development, for example [100]. Based on the differentially-expressed miRNA data presented in this study, we wanted to investigate (as a first-step approach) potential cellular pathways that may be regulated by the groups of miRNAs expressed more highly in progenitor, differentiated or hypertrophic chondrocytes. It must be stressed that the bioinformatics approach taken here has generated preliminary data that will require further validation once functional activity for these miRNAs has been confirmed. One attractive approach to confirm (or disprove) bioinformatics-based target gene data involves biochemically identifying target genes located within the RNA-induced silencing complex (RISC) followed by RNA-Seq analysis [101]; we are currently establishing this methodology in our laboratory.

For the purpose of this report, we have presented significantly enriched pathways related only to developmental processes that are known from *in vivo* and *in vitro* data to be important within the context of chondrogenesis. Other significantly enriched pathways were identified from our bioinformatics approach (i.e. related to cell cycle, transcription, apoptosis, cytoskeletal remodeling etc), but making any associations with other cellular processes at this stage would be over-interpreting the data presented in this study. With respect to those developmental pathways identified (**Tables 5** **,** **6** **,** **7** **,** **8**), a number of the same pathways were shown to be significantly enriched for each comparison analyzed (i.e. pathways related to TGFβ, BMP, FGF and IGF signaling). This is not surprising since these growth factors are known to be important in controlling many aspects of cartilage development and maintenance. Interestingly, the pathway involving activin A was also enriched for each comparison analyzed. Less is known about the function of this secreted factor in cartilage biology although there are reports that this TGF-β family member plays a role in developing as well as mature cartilage [102]–[104]. **Tables 6** **,** **7** **and** **8** shows that Hedgehog and Wnt signaling pathways [105], [106] may be regulated by miRNAs found to be more highly expressed in differentiated or hypertrophic chondrocytes. These pathways are particularly prominent in **Table 6** suggesting that miRs-138, 365 and 193b (**Table 2**) play a role in regulating these pathways to permit proper cartilage formation. VEGF-related signaling pathways were identified in the PC>DC and DC>HYP comparisons (**Tables 5** **and** **7**). Since VEGF plays a role during vascular invasion of hypertrophic chondrocytes to permit endochondral ossification [107], it is possible that specific miRNAs may function to inhibit these processes in cartilage regions that are not destined to be invaded by vessels and replaced by bone. On the other hand, pathway analysis revealed that miRNAs more highly expressed in hypertrophic chondrocytes may target the pathway involving IL-8 and angiogenesis (**Table 8**). This suggests that miRNAs in hypertrophic chondrocytes may regulate specific target genes to promote neovascularization and endochondral bone formation. Overall, these bioinformatics-based findings have generated some clues to suggest that certain developmental pathways may be regulated by specific miRNAs at a precise point in differentiation to control proper limb formation.

## Conclusions

This study has identified differentially-expressed miRNAs at a defined time point during human cartilage development of the embryonic limb. These findings should provide insights into miRNA-driven processes that are necessary to generate the formation of chondroprogenitor, differentiated or hypertrophic chondrocytes. Clinically, this work may be important for the design of miRNA-based tissue engineering strategies to promote endochondral bone repair or regeneration by enhancing hypertrophic differentiation and endochondral ossification processes, for example. Alternatively, miRNA based strategies to inhibit hypertrophic chondrocyte differentiation could be beneficial for generation of permanent articular cartilage tissue. It will also be interesting to investigate if dysregulation of miRNAs important in regulating developmental processes can cause skeletal defects such as osteoarthritis, chondrodysplasias or delayed endochondral fracture healing.

## Supporting Information

Figure S1 **Correlation of miRNA delta Ct values obtained from human microRNA OpenArray® screens using two different protocols**. Human lung RNA was prepared for the OpenArray® analysis using the manufacturer's protocol (protocol 2) or an adjusted protocol (protocol 1) to accommodate lower concentrations of sample RNA. See Materials and Methods section for a description of what aspects of the sample preparation protocol were changed.
(EPS)

Table S1 Genes predicted to be targeted by one or more miRNAs identified in Table 2 as being more highly expressed in PC compared to DC cells.
(XLSX)

Table S2 Genes predicted to be targeted by one or more miRNAs in Table 2 as being more highly expressed in DC compared to PC cells.
(XLSX)

Table S3 Genes predicted to be targeted by one or more miRNAs identified in Table 4 as being more highly expressed in DC compared to HYP cells.
(XLSX)

Table S4 Genes predicted to be targeted by one or more miRNAs identified in Table 4 as being more highly-expressed in HYP compared to DC cells.
(XLSX)
